# Dynamic Changes in Local Protein Synthetic Machinery in Regenerating Central Nervous System Axons after Spinal Cord Injury

**DOI:** 10.1155/2016/4087254

**Published:** 2016-06-07

**Authors:** Rahul Sachdeva, Kaitlin Farrell, Mary-Katharine McMullen, Jeffery L. Twiss, John D. Houle

**Affiliations:** ^1^Department of Neurobiology and Anatomy, Drexel University College of Medicine, Philadelphia, PA 19129, USA; ^2^Department of Biological Sciences, University of South Carolina, Columbia, SC 29208, USA

## Abstract

Intra-axonal localization of mRNAs and protein synthesis machinery (PSM) endows neurons with the capacity to generate proteins locally, allowing precise spatiotemporal regulation of the axonal response to extracellular stimuli. A number of studies suggest that this local translation is a promising target to enhance the regenerative capacity of damaged axons. Using a model of central nervous system (CNS) axons regenerating into intraspinal peripheral nerve grafts (PNGs) we established that adult regenerating CNS axons contain several different mRNAs and protein synthetic machinery (PSM) components in vivo. After lower thoracic level spinal cord transection, ascending sensory axons regenerate into intraspinal PNGs but axon growth is stalled when they reach the distal end of the PNG (3 versus 7 weeks after grafting, resp.). By immunofluorescence with optical sectioning of axons by confocal microscopy, the total and phosphorylated forms of PSMs are significantly lower in stalled compared with actively regenerating axons. Reinjury of these stalled axons increased axonal localization of the PSM proteins, indicative of possible priming for a subcellular response to axotomy. These results suggest that axons downregulate protein synthetic capacity as they cease growing, yet they retain the ability to upregulate PSM after a second injury.

## 1. Introduction

Regeneration of severed central nervous system (CNS) axons is one of the major challenges for recovery after spinal cord injury (SCI). Axons encounter various extracellular cues as they respond to the local environmental changes occurring after SCI and as they begin to regenerate, making rapid “decisions” distant from the neuron cell body. Numerous studies suggest that the intracellular response to extracellular cues ultimately promotes or inhibits axonal outgrowth [1, 2]. Following axotomy an injury signal is transported to the cell body and the distal segment of an axon undergoes Wallerian degeneration. The proximal segment is rapidly resealed and forms a new growth cone as a part of the initiation of a regenerative response [3]. It is known that axons rely, at least in part, on localized protein synthesis to fine-tune their spatiotemporal response to stimuli [4]. At least in the peripheral nervous system (PNS), local synthesis of proteins is required for communication between the injured axon and its soma [5], and growth cone formation after axotomy requires local protein synthesis in cultured neurons [6]. Recent work indicates that the microenvironment can alter translation in mature CNS axons [7]. Adult cortical axons in culture contain several hundred mRNAs related to axonal maintenance and show increased localization of mRNAs related to axonal targeting and synaptic function upon axotomy, indicative of enhanced capacity of axons to grow and form new synapses with targets [8]. Several studies show that mature mammalian axons retain their capacity to synthesize proteins [9–12]. We recently showed that, after spinal cord injury, CNS axons contain mRNAs and components of protein synthesis machinery (PSM) as they regenerate along a permissive environment of a peripheral nerve graft (PNG) [12]. PNGs promote robust regeneration of axons and functional recovery after spinal cord injury by providing a laminin-based, neurotrophin-rich environment that bypasses the growth inhibitory spinal cord environment that forms after SCI [13, 14]. Axonal growth beyond this supportive environment is greatly limited by growth inhibitory molecules such as chondroitin sulfate proteoglycans (CSPGs) of the extracellular matrix that severely limit the applicability of most therapeutic procedures targeting axon regeneration. Since axonal synthesis of proteins contributes to both neurite extension [15] and CSPG-mediated growth inhibition [2, 16], more studies are needed to expand the present day knowledge and provide opportunity for a targeted approach to regenerative therapies.

On-site protein synthesis provides considerable autonomy to the axons relative to the cell body and allows them to respond efficiently to local extracellular cues by modulating their local protein repertoire [2, 16, 17]. Assessing the translational capacity of axons during various stages of regeneration is an important step towards understanding possible mechanisms by which some neurons/axons exhibit “more effective” regeneration than others. Verma et al. [6] suggested that low levels of translational machinery in CNS axons compared to PNS axons might be related to reported differences in their intrinsic regenerative capacity. In this study we used a rat spinal cord transection injury and peripheral nerve grafting to provide a scenario where we could quantify PSM levels in CNS axons as they are growing into the PNG, when they stop growing, and when they are exposed to a second injury. We show that relative to an active regenerative state PSM levels decline when axons are no longer growing, yet subsequent to reinjury of 7-week (quiescent) axons there is a robust increase of PSM levels. Whether this increase in machinery cues the return to a growing state remains to be demonstrated but it is tempting to envision this as a signal that could be taken advantage of in future studies aiming to promote regeneration. This work highlights the need for further investigation of the potential for modulating intra-axonal translation to promote CNS regeneration.

## 2. Experimental Procedures

### 2.1. Spinal Cord Injury and Peripheral Nerve Grafting Procedures

Adult female Sprague Dawley rats (225–250 g, Charles River Laboratories International, Inc.) were used. All surgical procedures and postoperative care were approved by the Drexel Institutional Animal Care and Use Committee and followed the guidelines provided by the National Institutes of Health.

Spinal cord injury and peripheral nerve grafting procedures were performed as published previously [12]. Briefly, in 6 donor rats, predegenerated nerves for grafting were prepared by cutting the tibial nerve bilaterally 7 days prior to harvesting. This yielded 12 nerve segments for grafting into a set of recipient rats (*n* = 12). Recipient rats were anesthetized using 5% Isoflurane and maintained at 1–3% Isoflurane during the surgery. Following dorsal laminectomy, T12 spinal cord was transected using vacuum aspiration through a glass micropipette creating a 2-3 mm lesion cavity. Donor rats were anesthetized using Ketamine (60 mg/kg, Ketaset, Fort Dodge Animal Health, Fort Dodge, IA) and Xylazine (10 mg/kg, Anased, Lloyd Laboratories, Shenandoah, IA) and an 8–10 mm long segment of predegenerated tibial nerve was harvested. One end of the nerve was apposed to the middorsal caudal wall of the lesion cavity to facilitate the regeneration of ascending axons into the graft. The perineurium of this end of the graft was sutured to the dura mater to secure the graft in place. The dura was sutured close using 9–0 sutures. Perineurium of the distal end of the graft was secured by sutures to the muscle attached to vertebral processes rostral to the lesion, leaving this end unapposed to the spinal cord. All recipient rats underwent the same injury and grafting procedure and were divided into 3 groups (*n* = 4 per group). All rats received one injection of sustained release Buprenorphine (1.0 mg/kg, Zoopharm, Laramie, WY) and twice daily Cefazolin (25 mg/mL, Sandoz, Princeton, NJ) for one week. Beginning 3 days prior to grafting all graft recipient rats received daily subcutaneous Cyclosporine A (10 mg/kg, Teva Czech Industries, Sellersville, PA) for 2 weeks to prevent graft rejection, before changing to oral administration (1 mg/mL in the drinking water) for the remainder of the experiment.

*Group 1 (3 wk)*. Rats received no further intervention and were euthanized 3 weeks after injury and grafting to assess PSM levels in actively growing axons. 

*Group 2 (7 wk)*. Rats received no further intervention and were euthanized 7 weeks after injury and grafting to assess PSM levels in axons stalled at the distal end of a PNG.

*Group 3 (Reinjury)*. Rats received no further manipulation until 7 weeks after grafting when the rats were anesthetized and the distal end of the nerve graft was exposed and trimmed by 1 mm using microscissors. The perineurium of the “new” distal end was sutured to surrounding muscle. The rats received no further intervention and were sacrificed 2 weeks later, that is, 9 weeks after injury, to assess the effect of a second injury on PSM levels in stalled axons. We allowed 2 weeks after reinjury because we did not know the timeframe when a change in PSM might take place. We felt justified in examining differences between axons at different time periods after the initial injury since very low levels of PSM were detected within axons at 7 weeks (see Section 3) and without any additional manipulation no increase in levels would be expected at 9 weeks after the initial injury.

### 2.2. Tissue Preparation

All rats were euthanized using an intraperitoneal injection of Euthasol (390 mg/kg pentobarbital sodium and 50 mg/kg phenytoin sodium IP, Virbac, Fort Worth, TX) and were perfused transcardially with cold saline (0.9% NaCl) followed by 2% paraformaldehyde (PFA). The PNGs were carefully dissected out and postfixed in 2% PFA overnight at 4°C and then transferred to 30% sucrose in 0.1 M phosphate buffer solution for at least 72 hrs for cryoprotection. The PNGs were embedded in Tissue-Plus OCT compound (Fisher Scientific), cut longitudinally into 10 *μ*m thick sections, and mounted directly on Superfrost Plus slides (Fisher Scientific). The sections were dried overnight at room temperature and stored at −20°C.

### 2.3. Immunohistochemistry

For immunofluorescence, tissue sections were blocked for nonspecific reactions in 10% goat serum and 0.2% triton X-100 in 1x phosphate buffered saline (PBS). After 1 hr incubation in blocking solution, sections were incubated overnight in primary antibody (Ab) solution containing 5% goat serum and 0.2% triton X-100 in 1x PBS. The following primary Abs were used: mouse (ms) anti-neurofilament (160 kDa, 1 : 1000, Millipore) or rabbit (rb) anti-neurofilament M (145 kDa, 1 : 200, Millipore) to visualize axons, rb anti-SCG10 (1 : 500, Novus Biologicals) as a marker for regenerating axons, rb anti-4EBP1 (1 : 500, Cell Signaling Technology), rb anti-phospho-4EBP1^Thr37/46^ (p4EBP1, 1 : 100, Cell Signaling Technology), rb anti-phospho-S6 ribosomal protein^Ser235/236^ (1 : 100, Cell Signaling Technology), ms anti-eIF2*α* (1 : 100, Cell Signaling Technology), and rabbit anti-phospho-eIF2*α* ^Ser51^ (peIF2*α*, 1 : 50, Cell Signaling Technology). Sections were washed in 1x PBS and incubated for 2 hrs in the following secondary antibodies diluted in blocking buffer: goat (gt) anti-ms IgG Cy*™*3 (1 : 2000, Jackson Immunoresearch Laboratories Inc.) and gt anti-rb IgG Alexa Fluor® 488 conjugates (1 : 500, Life Technologies). Nuclei were stained by washing the sections in 1x PBS containing DAPI dilactate (Sigma). The sections then were washed in 1x PBS and cover-slipped using Cytoseal for imaging.

### 2.4. Imaging and Analysis of Axonal PSM

Imaging was performed using a confocal microscope (Zeiss LSM 700) and Zen software (Zeiss). All image acquisition parameters such as laser power, pinhole, PMT gain/offset, and pixel dwell time were matched within individual markers of PSM between the experimental groups. A PNG section from each rat reacted with all reagents except a primary Ab which was used as a reference for acquisition of background signals. Imaging parameters were set below those producing no signal with primary Ab control. Images were obtained at 63x magnification as three-dimensional z-stacks of 100 × 300 *μ*m in area generated by automated stitching of three individual 100 × 100 *μ*m tiles. z-stacks were acquired at 0.3 *μ*m step interval between planes with a total of 10–15 planes per stack spanning a depth of 3.0–4.5 *μ*m. The tile scans were taken at two comparable locations within the distal third of each nerve section and a total of 3 sections were analyzed per rat.

To quantify the intensity of intra-axonal signal, each z-stack was resolved into individual* XY* planes containing both neurofilament staining as an axonal marker and one of the markers of PSM. The RG2B plug-in (https://imagej.nih.gov/ij/plugins/rg2bcolocalization.html) on NIH ImageJ (https://imagej.nih.gov/ij/) was used to extract the pixels that colocalized between neurofilament stain and PSM markers. This was performed for each plane of the stack to isolate pixels representing axonal signal. The pixel intensity from each plane was then normalized to the neurofilament area in corresponding planes to account for the variability of number of axons per tile scan. The resultant values for each plane were averaged to get a mean value per z-stack.

### 2.5. Statistical Analyses

GraphPad Prism software was used for statistical analyses. Means across the multiple experimental groups were compared using one-way analysis of variance (ANOVA) followed by Holm-Šídák post hoc test for multiple comparisons. Information related to the *F* and *p* values for the individual ANOVA analyses is presented in Table 1. After multiple comparisons analyses, the graphs represent paired groups showing the fold change of fluorescence signal intensity and are presented as mean ± SD. The *p* value of ≤0.05 was considered as statistically significant.

## 3. Results

### 3.1. Timeline for Axonal Regeneration into the Grafts

**Figure 1:**
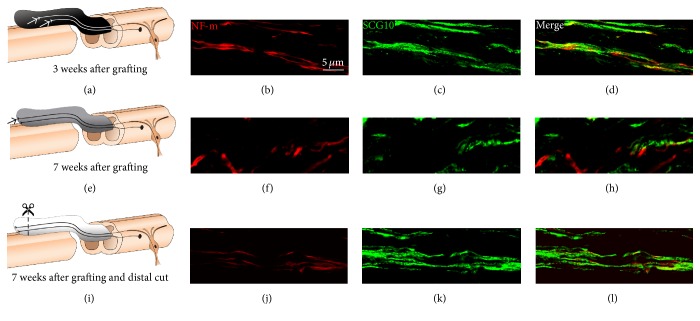
Experimental strategy and animal groups. All rats received a complete transection of the T12 spinal cord and immediate placement of PNGs of approximately 10 mm in length. (a)* Group 1* rats received no further intervention and were euthanized at 3 weeks after grafting when the majority of axons are in active regeneration phase (arrows) and growing towards the distal end of the PNG. (b, c) show actively growing axons coimmunolabeled for NF-m and SCG10, respectively. (d) shows the merge of the two panels. (e)* Group 2* rats received no further intervention and were euthanized at 7 weeks after grafting when axons had reached the unapposed distal end of the PNG (arrow) and stopped growing. (f, g) show axons “stalled” at the distal end that are coimmunolabeled for NF-m and for SCG10, respectively. (h) shows the merging of these panels. (i)* Group 3* rats received a second intervention at 7 weeks after grafting when the distal end of the PNG was exposed and trimmed by 1 mm (dashed line) to reinjure the “stalled” axons (i). The rats were euthanized 2 weeks later. (j, k) show reinjured axons at the distal graft end coimmunolabeled for NF-m and SCG10. (l) shows the merged image. The colors (black, gray, or white) of PNGs in (a), (e), and (i) correspond to the color of bar graphs for 3 weeks, 7 weeks, and 7-week reinjury, respectively, in Figures 2, 3, and 4. Scale bar = 5 *μ*m.

Complete transection of the spinal cord allows for the PNG apposition to a lesion cavity wall where all the axons are severed, thus creating an unambiguous model where only regenerating CNS axons would grow into the PNG. Another advantage that this model offers is that the axons usually grow in a linear arrangement along the length of the graft and allow easy visualization of the intra-axonal content in a considerable length of axon over a few optical planes. Axons were identified using neurofilament immunostaining for characterizing the time line for regeneration into the PNGs (Figures 1(b), 1(f), and 1(j)). Three weeks after grafting (Figures 1(a) and 1(b)), the majority of axons are present in the distal third of the nerve graft growing towards the unapposed end as shown. These represent the actively growing axons that are strongly immunopositive for SCG10 (Figure 1(c)) that is preferentially expressed in regenerating axons [18]. SCG10 is used here as an indication of a regenerated axon but we had insufficient material to perform quantitative assessment of SCG10 content between the 3 groups.

### 3.2. Regenerating Axons Downregulate PSM as Regeneration Ceases

**Figure 2 fig2:**
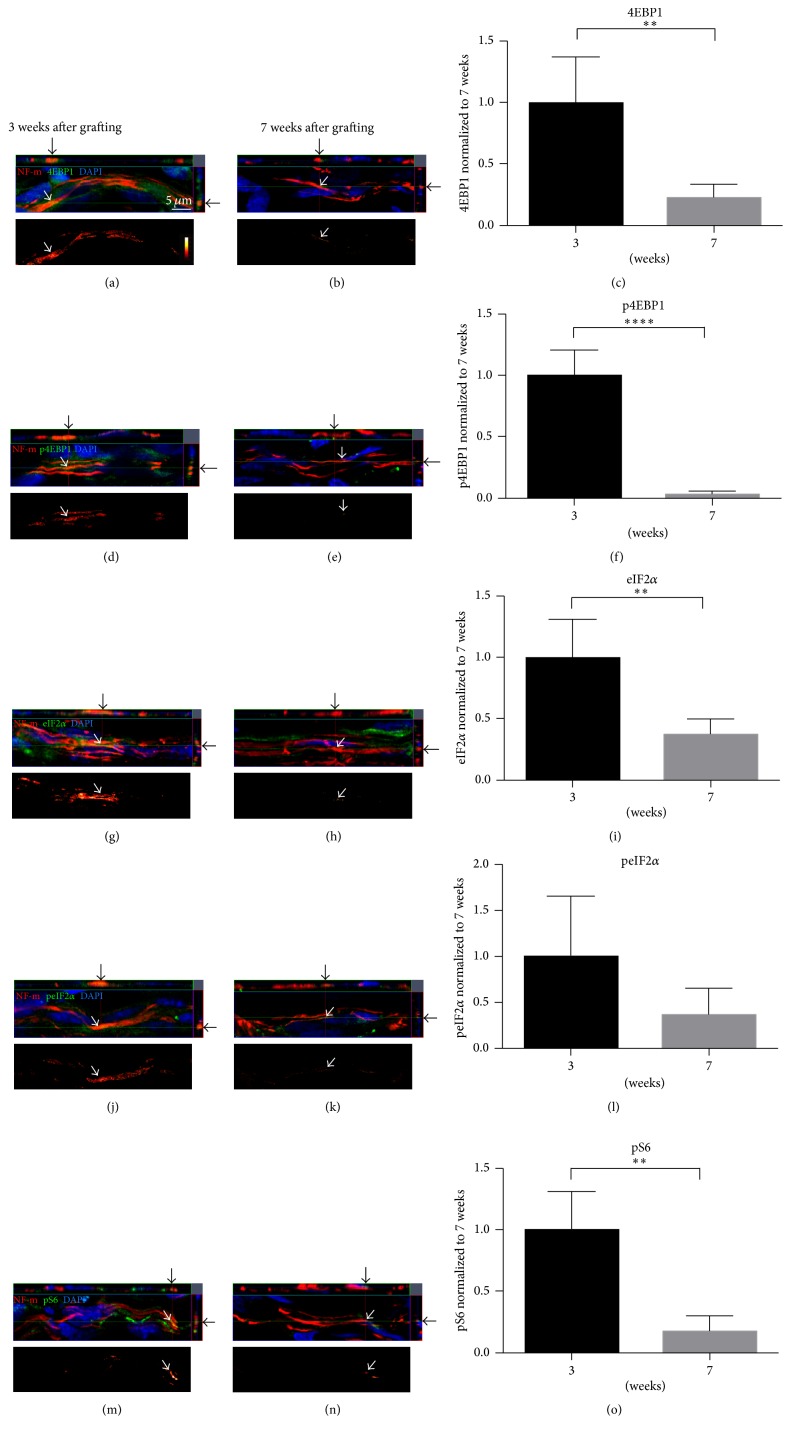
Intra-axonal PSM is downregulated when axonal growth “stalls.” Representative confocal images from ascending CNS axons immunolabeled for NF-m (red) in PNGs at 3 weeks (a, d, g, h, and m) and 7 weeks (b, e, h, k, and n). The image sets colabeled for 4EBP1 (a, b), p4EBP1 (d, e), eIF2*α* (g, h), peIF2*α* (j, k), and pS6 (m, n) are shown. Top panel of each image set represents exposure matched orthogonal projections showing* XY*,* XZ*, and* YZ* planes indicating axon-specific signal (arrows). Bottom panel of each image set shows the subtracted image with axon-only signal (see Section 2.4 for details) for each specific protein as indicated by an intensity spectrum. These projections were generated from 8 to 10 optical sections with 0.3 *μ*m z-step intervals. Scale bar = 5 *μ*m. Graphs (c), (f), (i), (l), and (o) show quantification of relative immunolabeling intensity for the subtracted axon-only signals for 4EBP1, p4EBP1, eIF2*α*, peIF2*α*, and pS6, respectively. Data are expressed as fold change in signal intensity compared with 3-week grafts ± SD. ^*∗*^ *p* ≤ 0.05, ^*∗∗*^ *p* ≤ 0.01, and ^*∗∗∗∗*^ *p* ≤ 0.001, using Holm-Šídák post hoc test.

Our unpublished observations indicate that regenerating axons reach the distal end of an 8–10 mm PNG during week 4 after grafting. At 7 weeks after grafting axons remain in a linear arrangement with abundant neurofilaments and SCG10 immunofluorescence in some but not all axon profiles (Figures 1(f) and 1(g)). Intra-axonal PSM was compared between actively growing axons (3 weeks) and the axons that have stopped growing (7 weeks). All markers used for PSM were detected in the axons and their levels were compared for axonal signal intensity from exposure matched images (Figure 2). Image sets (a)-(b), (d)-(e), (g)-(h), (j)-(k), and (m)-(n) of Figure 2 show axons at 3 and 7 weeks after grafting colabeled for NF and 4EBP1, p4EBP1, eIF2*α*, peIF2*α*, or pS6, respectively. The results are represented as the intensity of signal from the 7-week group normalized to the 3-week group. The levels of both total 4EBP1 (Figure 2(c)) and p4EBP1 (Figure 2(f)) were significantly lower in the 7-week group compared to the 3-week group (*p* < 0.01 and *p* < 0.001, resp.). No statistically significant change was observed in eIF2*α* and peIF2*α* levels between the two groups (Figures 2(i) and 2(l)). Finally, levels of pS6 were significantly lower in the 7-week axons compared to 3-week axons (*p* < 0.01, Figure 2(o)). These data indicate that axonal levels of PSM components, including the phosphorylated forms (4EBP1 and S6) decrease as the axons reach the end of the PNG and their growth stalls.

### 3.3. A Second Injury to the Axons Results in an Increase in Axonal PSM

**Figure 3:**
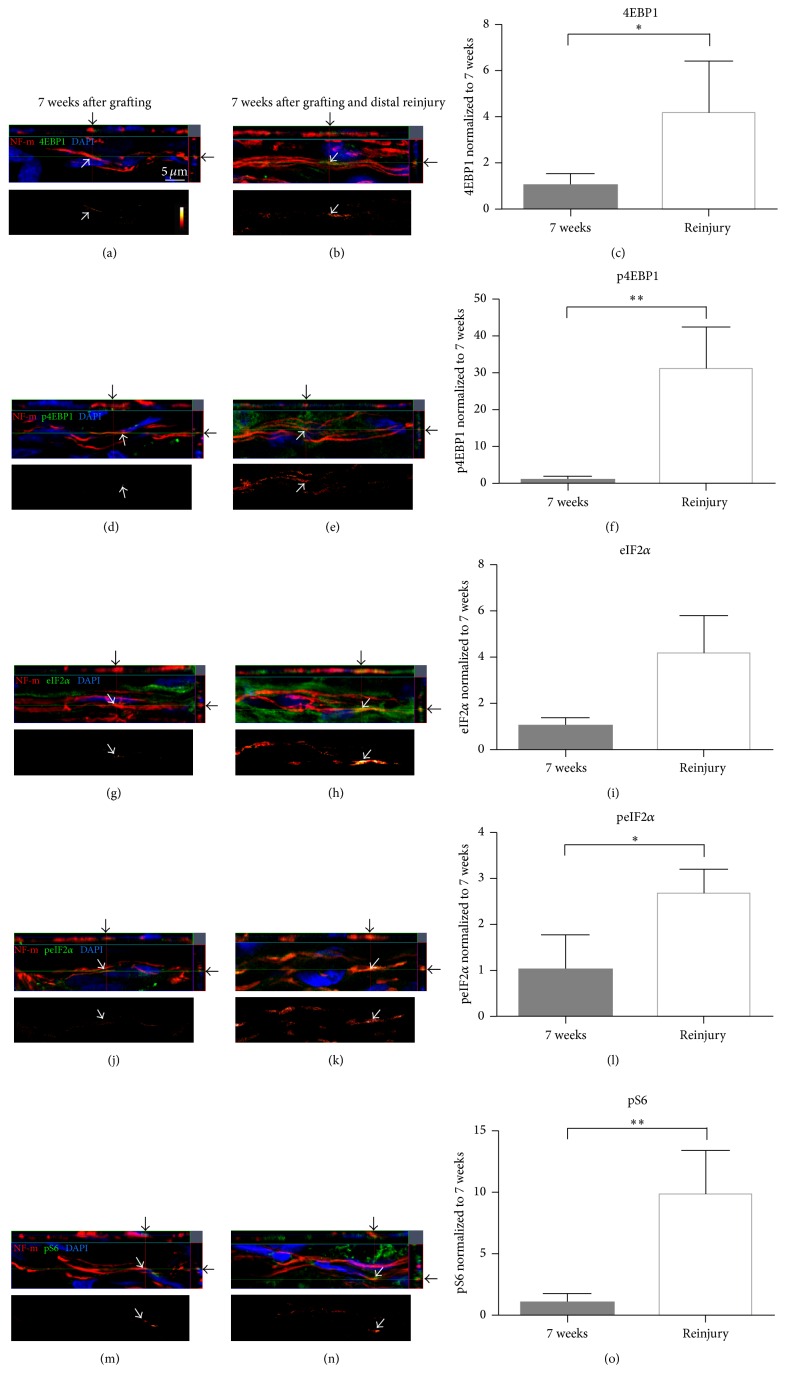
Stalled axons upregulate PSM upon reinjury. Representative confocal images from ascending CNS axons immunolabeled for NF-m (red) in PNGs at 7 weeks after grafting ((a), (d), (g), (h), and (m), the same as Figure 2) and the reinjury group (b, e, h, k, and n). The image sets colabeled for 4EBP1 (a, b), p4EBP1 (d, e), eIF2*α* (g, h), peIF2*α* (j, k), and pS6 (m, n) are shown. Similar to Figure 2, top panel of each image set represents exposure matched orthogonal projections showing* XY*,* XZ,* and* YZ* planes indicating axon-specific signal (arrows). Bottom panel of each image set shows the subtracted image with axon-only signal for specific protein as indicated by an intensity spectrum. Scale bar = 5 *μ*m. Graphs (c), (f), (i), (l), and (o) show quantification of relative immunolabeling intensity for the subtracted axon-only signals for 4EBP1, p4EBP1, eIF2*α*, peIF2*α*, and pS6, respectively. Data are expressed as fold change in signal intensity compared with 7-week grafts ± SD. ^*∗*^ *p* ≤ 0.05 and ^*∗∗*^ *p* ≤ 0.01, using Holm-Šídák post hoc test.

**Figure 4:**
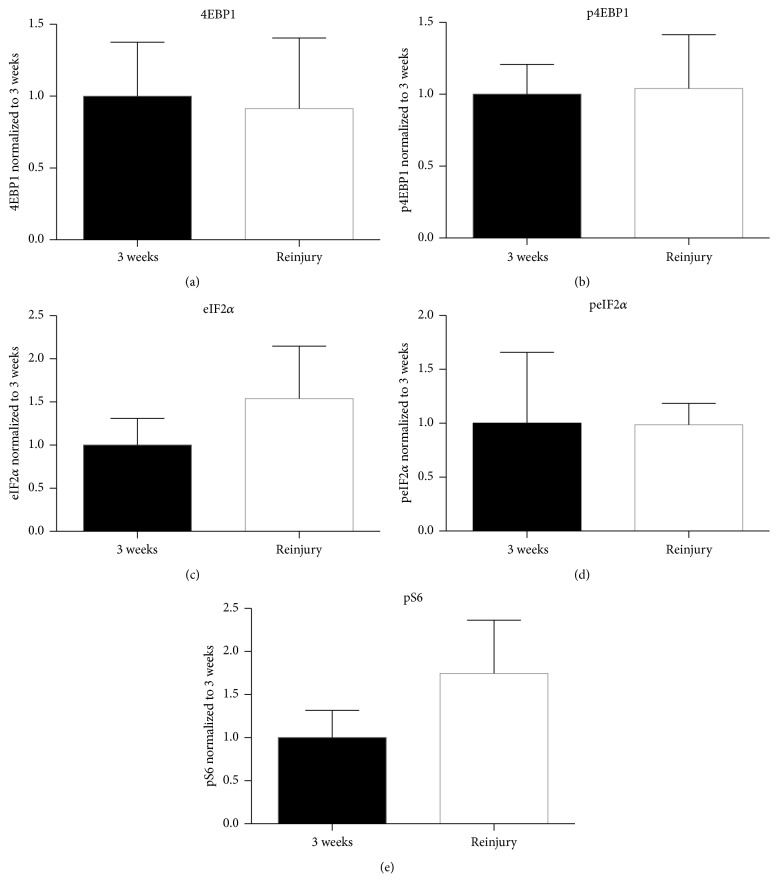
Translational machinery in reinjured and actively growing axons is comparable. Graphs (a)–(e) show quantification of relative immunolabeling intensity for the subtracted axon-only signals for 4EBP1, p4EBP1, eIF2*α*, peIF2*α*, and pS6, respectively. Data are expressed as fold change in signal intensity after reinjury at 7-week compared with 3-week PNGs ± SD, using Holm-Šídák post hoc test. No significant differences were seen between the two groups; however the levels of pS6 between the two groups trended towards significance (*p* = 0.07).

The unapposed distal end of PNG was cut (reinjured) at 7 weeks in Group 3 rats (Figure 1(i)) and the axonal PSM levels were compared to the 7-week nonreinjured/stalled axons of Group 2. SCG10 was observed in most axons that were examined 2 weeks after a second injury (Figure 1(k) [Fig fig2]). Image sets (a)-(b), (d)-(e), (g)-(h), (j)-(k), and (m)-(n) of Figure 3 show uninjured axons at 7 weeks after grafting and reinjured axons at 9 weeks (2 weeks after second injury) colabeled with 4EBP1, p4EBP1, eIF2*α*, peIF2*α*, and pS6, respectively. The data are represented as the signal intensity of axonal PSM in the reinjury group normalized to the 7-week nonreinjured group. Axonal 4EBP1 (Figure 3(c)) and p4EBP1 (Figure 3(f)) levels were significantly higher after a reinjury with p4EBP1 compared to uninjured axons at 7 weeks after grafting. The levels of axonal eIF2*α* (Figure 3(i)) and peIF2*α* (Figure 3(l)) were significantly higher in response to the axonal reinjury. Similarly, the levels of axonal pS6 were also increased in response to the second injury (Figure 3(o)). Finally, the levels of all PSM molecules tested were statistically equivalent in the reinjury group compared to those in the 3-week group (Figures 4(a)–4(e)). Interestingly, reinjury caused an upregulation in the levels of axonal pS6 that approached towards a statistically significant increase (*p* = 0.07, Figure 4(e)).

## 4. Discussion

The role of locally translated proteins in axon growth and path finding during PNS development or regeneration is well known [7, 19]. What remains a mystery is how it is utilized to promote efficient CNS regeneration. Not only is the regenerative capacity of CNS far less than in the PNS but also CNS regeneration failure is more pronounced as the neurons mature [20]. The Fawcett laboratory indicated that mature CNS neurons lose the capacity for axonal protein synthesis which likely contributes to regeneration failure after CNS trauma [6]. While it is known that adult sensory neurons retain the capacity for intra-axonal translation [11, 21], only recently is there data suggesting that regenerating CNS axons localize local mRNAs and PSM [12].

### 4.1. Axonal Protein Synthesis Is an Important Target Mechanism for Enhancing CNS Regeneration

The basis for this study comes from our previous finding that regenerating axons show the potential for local translation during the phase of active regeneration, which, in some cases, is comparable to regenerating PNS axons [12]. The experiments here address the question of whether the local translational machinery of axons changes with their regenerative state and/or in response to extrinsic cues such as a second injury. Consistent with earlier studies that have shown that ribosomes localize both in dendrites [22] and in axons [23], we showed that axons are positive for phosphorylated ribosomal protein S6, a component of 40S ribosomal subunit. Phosphorylated S6 is more frequently associated with translationally active ribosomes (as opposed to subunits), so it has been used as a surrogate measure of translational activity. In contrast the absolute levels of translation factors vary independently of ribosome levels, so for those we measured the total proteins. Although the mechanistic role of S6 phosphorylation in regenerative growth is unclear, a number of studies indicate that higher levels of phosphorylated S6 provide selective advantage to 40s subunits for recruitment in polysomes [24–26]. S6 phosphorylation is one of the critical effector mechanisms of mammalian target of Rapamycin (mTOR) that strongly mediates the regenerative response of injured neurons [27, 28]. Another important downstream target of mTOR is 4EBP1 that sequesters the initiation factor eIF4E to control the initiation of cap dependent translation. Phosphorylation of 4EBP1 disrupts its binding from eIF4E and allows it to bind the mRNA cap to initiate translation [29]. Furthermore, translation initiation is tightly regulated by eIF2, whose *α* subunit, when phosphorylated, reduces the overall rate of protein synthesis but increases translation of certain specific mRNAs such as those associated with integrated stress response [30], including at least some mRNAs in PNS axons [31].

We first compared the levels of these factors between axons that are actively growing (3 weeks) and the axons that have stopped growing (7 weeks) upon reaching the sealed end of the distal unapposed PNG. We measured a decrease in PSM levels in the 7-week axons compared to 3-week axons, further strengthening the premise that local translation machinery may be most useful during axon growth and pathfinding and that it is probably downregulated after axons come to a stop.

### 4.2. Reinjury Upregulates Axonal PSM

The number of patients with chronic SCI is increasing steadily due to improved acute posttrauma care [32] such that subjects have a near normal life span. From a clinical standpoint, whether chronically injured axons can be reprimed for regeneration is a largely unexplored question. To address whether “translationally quiescent” axons can be “coaxed” towards regeneration, we axotomized axons by trimming the distal end of the PNG 7 weeks after grafting. Although different in principle, this may loosely mimic the “conditioning lesion” approach for improving CNS regeneration [33], which also correlates with increase in translation machinery [6]. This second injury to the axons showed a remarkable upregulation in all the markers of PSM indicative of their being primed for regeneration, raising an exciting possibility that CNS neurons retain their capacity for local translation for relatively longer timepoints after injury and that this capacity may be exploited for regeneration. Surprisingly, in response to reinjury, we saw an upregulation in peIF2*α*, which is known to be a general repressor of protein synthesis. While phosphorylation of eIF2*α* is known to reduce overall mRNA translation, it is not sufficient for repressing translation of all mRNAs in a cell. There are certain transcripts that escape this mechanism and, in fact, these are preferentially translated with increased eIF2*α* phosphorylation [34]. The protein expression from individual genes during a global reduction in mRNA translation can be achieved by nonconventional translation mechanisms [34]. A hybridization array analysis showed that approximately 2.5% of mouse liver mRNAs are upregulated during eIF2*α* phosphorylation [35]. One example among others is translation of activating transcription factor (ATF) 4, a transcriptional regulator of genes involved in metabolism and nutrient uptake, the redox status of cells, and the regulation of apoptosis, leading to an integrated stress response [36, 37]. Interestingly, ATF4 is also synthesized locally in adult mammalian axons in response to extracellular amyloid-*β* peptide [38]. More experimentation will be needed to answer whether similar stress mechanisms are operational when axons are reinjured.

## 5. Conclusions

While we are beginning to understand the intricacies of mechanisms involved in crosstalk between the axon and the environment, these results suggest an important avenue of research to modulate local translation for efficient CNS regeneration. While upregulation of PSM indicates the increase in translation capacity, more studies will be needed to test how local levels of specific mRNA transcripts/proteins that are related to neurite extension or collapse change with extracellular stimuli. Recently we provided evidence for intra-axonal transport of mRNAs related to axonal growth after SCI [12]. This is of particular importance since local protein synthesis is important not just for neurite/growth cone extension, but also for growth inhibition [2]. A comprehensive understanding of these pathways can help develop potential therapeutic targets to improve regeneration after SCI.

## Figures and Tables

**Table 1:** One-way ANOVA *F* and *p* values comparing fold change in signal intensity for target proteins measured across each group (3-week, 7-week, reinjury).

Protein	*F* value	*p* value
4EBP1	5.584	0.027
p4EBP1	21.35	0.0004
eIF2*α*	8.591	0.0082
peIF2*α*	2.801	0.113
pS6	14.83	0.0014
